# International versus national growth charts for identifying small and large-for-gestational age newborns: A population-based study in 15 European countries

**DOI:** 10.1016/j.lanepe.2021.100167

**Published:** 2021-07-15

**Authors:** Alice Hocquette, Mélanie Durox, Rachael Wood, Kari Klungsøyr, Katarzyna Szamotulska, Sylvan Berrut, Tonia Rihs, Theopisti Kyprianou, Luule Sakkeus, Aline Lecomte, Irisa Zile, Sophie Alexander, Jeannette Klimont, Henrique Barros, Miriam Gatt, Jelena Isakova, Béatrice Blondel, Mika Gissler, Jennifer Zeitlin

**Affiliations:** a Université de Paris, CRESS, Obstetrical Perinatal and Pediatric Epidemiology Research Team, EPOPé, INSERM, INRA, F-75004 Paris, France; b Public Health Scotland, Edinburgh and University of Edinburgh, Edinburgh, Scotland; c Division of Mental and Physical Health, Norwegian Institute of Public Health, Bergen, Norway and Department of Global Public Health and Primary Care, University of Bergen, Norway; d Institute of Mother and Child, Department of Epidemiology and Biostatistics, Warsaw, Poland; e Federal Statistical Office FSO, Neuchâtel, Switzerland; f Health Monitoring Unit, Ministry of Health, Nicosia, Cyprus; g Estonian Institute for Population Studies, Tallinn University, Tallinn, Estonia; h Department of Population Health, Luxembourg Institute of Health, Luxembourg; i The Centre for Disease Prevention and Control of Latvia, Riga, Latvia; j Université Libre de Bruxelles, School of Public Health, Perinatal Epidemiology and Reproductive health Unit, Brussels, Belgium; k Statistics Austria, Vienna, Austria; l University of Porto Medical School, Department of Public Health, Forensic Sciences and Medical Education, Porto, Portugal; m Directorate for Health Information and Research, National Obstetric Information Systems (NOIS) Register, Tal-Pietà, Malta; n Institute of Hygiene, Health Information Centre, Health Statistics Department, Vilnius, Lithuania; o THL Finnish Institute for Health and Welfare, Information Services Department, Helsinki and Karolinska Institute, Department of Neurobiology, Care Sciences and Society, Stockholm, Sweden

**Keywords:** fetal growth, small for gestational age, large for gestational age, fetal growth charts

## Abstract

**Background:**

To inform the on-going debate about the use of universal prescriptive versus national intrauterine growth charts, we compared perinatal mortality for small and large-for-gestational-age (SGA/LGA) infants according to international and national charts in Europe.

**Methods:**

We classified singleton births from 33 to 42 weeks of gestation in 2010 and 2014 from 15 countries (*N* = 1,475,457) as SGA (birthweight <10th percentile) and LGA (>90th percentile) using the international Intergrowth-21st newborn standards and national charts based on the customised charts methodology. We computed sex-adjusted odds ratios (aOR) for stillbirth, neonatal and extended perinatal mortality by this classification using multilevel models.

**Findings:**

SGA and LGA prevalence using national charts were near 10% in all countries, but varied according to international charts with a north to south gradient (3.0% to 10.1% and 24.9% to 8.0%, respectively). Compared with appropriate for gestational age (AGA) infants by both charts, risk of perinatal mortality was increased for SGA by both charts (aOR[95% *confidence interval* (CI)]=6.1 [5.6–6.7]) and infants reclassified by international charts from SGA to AGA (2.7 [2.3–3.1]), but decreased for those reclassified from AGA to LGA (0.6 [0.4–0.7]). Results were similar for stillbirth and neonatal death.

**Interpretation:**

Using international instead of national charts in Europe could lead to growth restricted infants being reclassified as having normal growth, while infants with low risks of mortality could be reclassified as having excessive growth.

**Funding:**

InfAct Joint Action, CHAFEA Grant n°801,553 and EU/EFPIA Innovative Medicines Initiative 2 Joint Undertaking ConcePTION grant n°821,520. AH received a PhD grant from EHESP.

**Research in context**

**Evidence before this study**

The Intergrowth-21st project published prescriptive international intrauterine and newborn growth charts in 2014, launching a vigorous debate about whether these charts should be used in clinical practice and research for the identification of small and large for gestational age infants (SGA and LGA) or whether local charts should be preferred. To review the papers evaluating these charts we searched PubMed for comparisons of the Intergrowth-21st charts with other local charts published from September 2014 to February 2020, combining the search terms “intergrowth” AND “fetal/intrauterine growth OR fetal/intrauterine growth restriction OR fetal/intrauterine growth retardation OR small for gestational age OR birthweight OR low birthweight OR large for gestational age OR macrosomia OR references OR standards OR growth charts OR growth curves OR biometric measures OR anthropometry”. Results from this literature review show that local or customised charts more accurately described the birthweight distribution and the mortality risks associated with low and high birthweight than the Intergrowth-21st charts in many settings. These studies have been single-country studies and international comparisons of the Intergrowth-21st charts are lacking.

**Added value of this study**

This study adds to the scientific literature by comparing the Intergrowth-21st newborn charts with national charts customised to each country's population in 15 European countries, making it possible to assess the consequences of using one universal chart versus country specific charts in an international context. The study uses routine population data on birthweight from 1.5 million births in European countries participating in the Euro-Peristat network. We find large differences in the prevalence of both SGA and LGA infants between international and national charts, with a strong north to south gradient when using international charts, demonstrating the major impact of the choice of chart on the comparative assessment of the burden of fetal growth anomalies by country and their relative rankings. Further, we show that births reclassified by the international chart from SGA to appropriate for gestational age (AGA) had over two-fold higher risks of perinatal mortality, whereas births reclassified from AGA to LGA had lower risk.

**Implications of all the available evidence**

Our results corroborate previous comparative single-country studies evaluating the Intergrowth-21st intrauterine growth charts. They provide further evidence in favor of using national or local growth charts for monitoring growth during pregnancy and at birth and suggest that physiological differences in population anthropometric characteristics should be taken into consideration when constructing growth charts. Moreover, our study sheds new light on the capacity of the Intergrowth-21st charts to identify SGA and LGA infants at risk of fetal and neonatal mortality in a European context; it illustrates limitations at both extremes of the birthweight spectrum in some settings which may create risks of underestimating SGA births and overestimating LGA births. All these elements do not provide support for the use of the Intergrowth-21st international chart for defining SGA and LGA at birth in Europe.

Alt-text: Unlabelled box

## 1 Introduction

Restricted and excessive growth are severe pregnancy complications associated with short and long-term adverse health outcomes. Fetal growth restriction, defined by insufficient growth in relation to the fetus’ genetic potential [1,2], is associated with risks of stillbirth and neonatal death, major neonatal morbidity, neuro-developmental and metabolic disorders [3], [4], [5]. Excessive growth, a complication of gestational diabetes, is also associated with fetal and neonatal death as well as hypoxic ischaemic encephalopathy, shoulder dystocia and childhood obesity [6], [7], [8], [9], [10]. While restricted and excessive growth are defined in relation to the fetus’ genetic potential, proxies based on weight are used in clinical practice and research. Small-for-gestational-age (SGA) is commonly defined as a birthweight under the 10th percentile and large-for-gestational-age (LGA) as a birthweight over the 90th percentile. While there is a broad consensus on these thresholds [1,10,11], there is an on-going debate about which growth charts should be used and, in particular, whether charts should be universal or specific to national populations.

In line with the World Health Organization charts for children project [12], the Intergrowth-21st project developed intrauterine growth charts based on the assumption that fetal growth is similar across diverse geographical settings as long as nutrition and access to health care are guaranteed and environmental constraints on growth are low [13], [14], [15]. Others claim that the physiological characteristics of each population are essential for defining risk and that national charts are more appropriate [16], [17], [18], [19]. Proponents of national charts point to studies showing the impact of geographic and ethnic origin on birthweight [17], [18], [19], while proponents of using a universal chart argue that population differences are minimal and that international norms are needed to assess deviation from normal growth [20]. This debate is of particular relevance in an international context for studies investigating differences between countries in the prevalence of SGA or LGA births or developing protocols and synthesising evidence across multiple settings.

The objective of this study is to compare the capacity of international neonatal charts, as proposed by the Intergrowth-21st project [13], and national charts customised to each country[21,22] to identify newborns at risk of perinatal mortality in 15 European countries. The European context is of interest given geographically proximate countries with similar standards of living, universal health insurance for pregnant women, but population differences in adult height and weight which may affect fetal size and corresponding thresholds for defining sub-optimal growth [23,24].

## 2 Methods

This study was undertaken by the Euro-Peristat network to underpin recommendations for selecting growth charts in the ConcePTION project, a European consortium on medications during pregnancy and breastfeeding. The Euro-Peristat network, constituted in 1999, aims to monitor and evaluate the health and care of pregnant women and babies in Europe based on national population data on perinatal health indicators [25,26].

### 2.1 Data source

The data source is a network study on intrauterine growth conducted in 2016–2017 which included 15 countries (Austria, Belgium, Cyprus, Estonia, Finland, France, Latvia, Lithuania, Luxemburg, Malta, Norway, Poland, Portugal, Scotland and Switzerland). Individual-level information was collected on five variables (birthweight, gestational age at birth, infant sex, vital status at birth (termination of pregnancy, stillbirth, livebirth), and neonatal death before 28 days of life) for all singleton births in the years 2010 and 2014. Data came from birth registers, civil registration systems and routine surveys (see Appendix A). Inclusion criteria, based on Euro-Peristat definitions, were a gestational age of at least 22 weeks of gestation or, if gestational age was missing, birthweight of at least 500 g. Gestational age was requested in complete weeks of gestation (e.g. a birth at 37 weeks and 6 days of gestation was recorded with a gestational age of 37 weeks). The definition of gestational age was the final estimate in the obstetrical records at birth.

Most countries provided data for their whole population for the given years, except for France where data come from a national survey including all births during a one-week period in all maternity hospitals in France. France and Poland provided information on stillbirths, but not on neonatal deaths since they weren't collected in the French Perinatal Survey and they couldn't be linked for the Polish data. France and Poland provided data for the year 2010 only, Portugal and Switzerland provided data for the years 2010 and 2013, and Cyprus provided data from 2007 to 2013 to allow for larger sample sizes in this small country.

### 2.2 Ethical approvals

This study uses a sub-set of Euro-Peristat's core variables, which include no indirect or direct personal identifiers. Data are provided to Euro-Peristat in accordance with each data provider's regulations for data use. The procedures for obtaining and maintaining the Euro-Peristat core indicator database were authorised by the French Advisory Committee on Use of Health Data in Medical Research (N°17–048, 30/03/17) and the French National Commission for Data Protection and Liberties (CNIL, DR.−2019–089, 26/03/19).

### 2.3 Study population

Among the 1496,321 singleton births in the 15 countries during the study period, we included live births and stillbirths from 33 to 42 weeks of gestation because the Intergrowth-21st newborn charts use these gestational age limits (*N* = 1477,840). We excluded terminations of pregnancy when it was possible to distinguish them from stillbirths in the dataset (*N* = 5); this was not possible in Belgium, Cyprus and Luxemburg (in 2010 only). Newborns with undetermined or unknown sex and with missing data on birthweight or gestational age were excluded (*N* = 2378). Missing data constituted less than 1% of all data, except for Luxembourg (1.8%). The final sample included 1475,457 births from 15 countries with data on stillbirths and 1062,154 births from 13 countries with data on neonatal deaths and stillbirths.

### 2.4 Outcomes

The study's principal outcomes were stillbirth, neonatal mortality and extended perinatal mortality (stillbirth or neonatal death). Countries have different lower gestational age limits for recording stillbirth [27], but this does not affect births at 33 weeks of GA and over which are registered in all countries. Neonatal death was defined as death before 28 days after a live birth. Rates were calculated per 1000 total births for stillbirth and extended perinatal mortality, and per 1000 live births for neonatal mortality.

### 2.5 Defining SGA and LGA births

#### 2.5.1 International prescriptive charts

To define SGA and LGA by international charts, we used the Intergrowth-21st standards for newborn weight [13]. These charts are part of a suite of charts developed by the Intergrowth 21st project for monitoring intrauterine growth from a sample of rigorously selected low-risk pregnancies from 8 countries (Brazil, Italy, Oman, UK, USA, China, India, and Kenya) [13,14]. Selection criteria included medical and obstetrical history, socio-demographic and behavioural (nutrition, smoking) characteristics, health service accessibility and current pregnancy complications. The newborn weight chart distinguishes boys and girls and covers births from 33 weeks of gestation up to 42 weeks. Centiles were fitted using fractional polynomials assuming a skew t distribution with four parameters (mean, standard deviation, skewness and kurtosis). Its published values are expressed in exact weeks (specifying the number of weeks and days; for example, 28 exact weeks corresponds to 28+0 days as opposed to 28 completed weeks which covers 28+0 to 28+6 days) [28]. To adapt to our data in completed weeks, we used the midpoint weight for each week.

#### 2.5.2 National descriptive charts based on the customised chart methodology

The national charts were modelled based on the customised chart methodology developed by Gardosi et al. [16]. Customised charts are widely used in the international literature on growth restriction and were adapted by Mikolajczyk et al. [21]. and others[22,29] for use at the country-level. The customised chart's principle is based on the calculation of an individual ideal birthweight at 40 weeks of gestation taking into consideration factors which physiologically affect growth (fetal sex, maternal height, pre-pregnancy weight, parity and ethnicity). To transpose this ideal birthweight to each week of gestation, Hadlock's growth trajectory (expressing estimated fetal weight by gestational age) is used to model individual intrauterine growth trajectories [30]. Assuming a normal distribution, the 10th and 90th percentiles are calculated as a proportion of this individual trajectory using a constant coefficient of variation (calculated as standard deviation over mean of birthweight at 40 weeks of gestation). For our study, in line with previous applications of this model on the country level [21,22], we used each country's mean birthweight and coefficient of variation at 40 weeks of gestation to create national charts for girls and boys separately.

Equations for the national charts 50th percentile (Eqn 1), 10th percentile (Eqn 2) and 90th percentile (Eqn 3) are:

(1) $$P_{50}=\frac{\exp\left(0.578+0.332w-0.00354w^{2}\right)\times m_{C}}{m_{H}}$$

(2) $$P_{10}=P_{50}\times \left(1-1.28\times \frac{s_{c}}{m_{C}}\right)$$

(3) $$P_{90}=P_{50}\times \left(1+1.28\times \frac{s_{c}}{m_{C}}\right)$$

Where: m_C_ is the mean birthweight at 40 weeks of gestation of the country (for boys and girls separately), m_H_ is the mean birthweight at 40 completed weeks of gestation as derived from Hadlock's study sample by Mikolajczyk et al.[21] (3705 g), s_C_ is the standard deviation of birthweight at 40 weeks of gestation of the country (for boys and girls separately) and w is gestational age expressed in exact weeks. The country-specific coefficients for the models are provided in Supplementary Table 1.

### 2.6 Analysis strategy

First, we compared the prevalence of SGA and LGA infants in each country according to the international and national charts[13,21] and assessed geographic patterns with maps as there are known gradients of birthweight in Europe from north to south [31]. Second, we classified our sample by both charts as: (1) SGA according to both charts; (2) SGA according to the international chart only; (3) SGA according to the national chart only; (4) AGA according to both charts; (5) LGA according to the international chart only; (6) LGA according to the national chart only; and (7) LGA according to both charts. We compared stillbirth, neonatal mortality and extended perinatal mortality rates by this classification and then derived adjusted odds ratios (aOR) using a multi-level logistic regression to take into consideration the clustering of births within countries. We adjusted our model on sex, but not on gestational age since it is an intermediate factor on the pathway between growth restriction and perinatal death (see directed acyclic graph in supplemental Figure 1). However, we carried out sub-group analysis for term births (37 weeks of gestation and over). As our outcomes are rare, odds ratios approximate relative risks.

All analyses were performed using Stata 14.0 [32].

### 2.7 Role of the funding source

The funders of the study had no role in study design, data collection, data analysis, data interpretation, or writing of the report. The corresponding author had full access to all the data in the study and AH and JZ had final responsibility for the decision to submit for publication.

## 3 Results

There was a wide range in the number of total births from 7984 in Malta to 398,764 in Poland (Table 1). The overall stillbirth rate was 1.8 per 1000 total births (95% confidence interval (CI): 1.8 to 1.9, 15 countries) with variation from 1.3 stillbirths per 1000 (CI: 1.1 to 1.4) in Portugal to 2.9 per 1000 (CI: 2.4 to 3.4) in Latvia. There were 0.8 neonatal deaths per 1000 live births (CI: 0.7 to 0.8, 13 countries) ranging from 0.2 per 1000 (CI: 0.1 to 0.7) in Luxembourg to 1.5 per 1000 (CI: 1.1 to 1.9) in Latvia. Extended perinatal deaths were 2.5 per 1000 total births (CI: 2.4 to 2.6, 13 countries), with a range from 1.6 (CI: 1.1 to 2.5) in Luxembourg to 4.8 (CI: 3.5 to 6.5) in Malta.

**Table 1:** Total births in the study sample and stillbirth and neonatal mortality rates between 33 and 42 weeks of gestation by country.

Country	Total births	Stillbirth rate		Neonatal mortality rate		Extended perinatal mortality rate	
		N	‰ total birth [CI 95%]	n	‰ live births[CI 95%]	n	‰ total births [CI 95%]
Austria	153,410	258	1.7	92	0.6	350	2.3
			[1.5; 1.9]		[0.5; 0.7]		[2.1; 2.5]
Belgium	71,988	156	2.2	70	1.0	226	3.1
			[1.9; 2.5]		[0.8; 1.2]		[2.8; 3.6]
Cyprus	20,290	39	1.9	18	0.9	57	2.8
			[1.4; 2.6]		[0.6; 1.4]		[2.2; 3.6]
Estonia	28,284	57	2.0	24	0.9	81	2.9
			[1.6; 2.6]		[0.6; 1.3]		[2.3; 3.6]
Finland	114,610	161	1.4	82	0.7	243	2.1
			[1.2; 1.6]		[0.6; 0.9]		[1.9; 2.4]
France	14,539	25	1.7	–	–	–	–
			[1.2; 2.5]				
Latvia	39,166	112	2.9	57	1.5	169	4.3
			[2.4; 3.4]		[1.1; 1.9]		[3.7; 5.0]
Lithuania	57 024	138	2.4	84	1.5	222	3.9
			[2.0; 2.9]		[1.2; 1.8]		[3.4; 4.4]
Luxembourg	12,854	18	1.4	3	0.2	21	1.6
			[0.9; 2.2]		[0.1; 0.7]		[1.1; 2.5]
Malta	7984	19	2.4	19	2.4	38	4.8
			[1.5; 3.7]		[1.5; 3.7]		[3.5; 6.5]
Norway	116,603	239	2.1	81	0.7	320	2.7
			[1.8; 2.3]		[0.6; 0.9]		[2.5; 3.1]
Poland	398,764	826	2.1	–	–	–	–
			[1.9; 2.2]				
Portugal	177,013	225	1.3	84	0.5	309	1.7
			[1.1; 1.4]		[0.4; 0.6]		[1.6; 2.0]
Scotland	107,791	230	2.2	75	0.7	305	2.8
			[1.9; 2.4]		[0.6; 0.9]		[2.5; 3.2]
Switzerland	155,137	217	1.4	122	0.8	339	2.2
			[1.2; 1.6]		[0.7; 0.9]		[2.0; 2.4]
Total	1,475,457	2720	1.8	811	0.8	2680	2.5
			[1.8; 1.9]		[0.7; 0.8]		[2.4; 2.6]

NOTE: Combined data from the years 2010 and 2014, except Cyprus (2007–2013), Poland and France (2010 only) and Portugal and Switzerland (2010, 2013).

The proportions of SGA and LGA based on national charts were close to the 10% expected values, with a minimum of 8.5% in Cyprus to a maximum of 10.6% in France for SGA, and from 10.3% in Latvia to 14.8% in Malta for LGA (Table 2). However, these proportions varied markedly when using the international charts: from 3.0% in Estonia to 10.1% in Portugal for SGA and from 8.0% in Portugal to 24.9% in Estonia for LGA. Differences in prevalence between the international and national charts were up to −6.7% for SGA prevalence and to 14.3% for LGA prevalence in Estonia. These discrepancies were geographically patterned, with a lower prevalence of SGA in the north and a higher prevalence of SGA in the south, and higher prevalence of LGA in the north and lower prevalence of LGA in the south when using the international chart (Figure 1a and Figure 1b).

**Table 2:** Prevalence of small and large for gestational age births in European countries according to international and national charts.

Country	Total births (N)	International			National		
		SGA (%)	AGA (%)	LGA (%)	SGA (%)	AGA (%)	LGA (%)
Austria	153,410	5.9	80.0	14.1	10.0	78.4	11.6
Belgium	71,988	8.2	80.1	11.7	10.1	77.8	12.1
Cyprus	20,290	7.7	81.5	10.8	8.5	77.4	14.1
Estonia	28,284	3.0	72.1	24.9	9.7	79.7	10.6
Finland	114,610	3.9	73.0	23.0	10.1	78	11.9
France	14,539	8.8	80.5	10.7	10.6	78.3	11.2
Latvia	39,166	4.0	72.8	23.2	10.1	79.6	10.3
Lithuania	57 024	4.4	75.2	20.5	9.7	79.3	11.1
Luxembourg	12,854	6.5	81.0	12.5	9.4	77.8	12.8
Malta	7984	8.6	80.8	10.6	9.6	75.6	14.8
Norway	116,603	3.9	72.8	23.3	10.4	78.5	11.1
Poland	398,764	6.7	77.0	16.3	9.3	79.3	11.4
Portugal	177,013	10.1	81.9	8.0	9.0	78.5	12.4
Scotland	107,791	6.5	75.1	18.4	10.1	78.6	11.3
Switzerland	155,137	6.4	80.4	13.2	9.6	78.5	11.9
Total	1475 457	6.4	77.5	16.1	9.7	78.7	10.7

**Fig. 1a:**
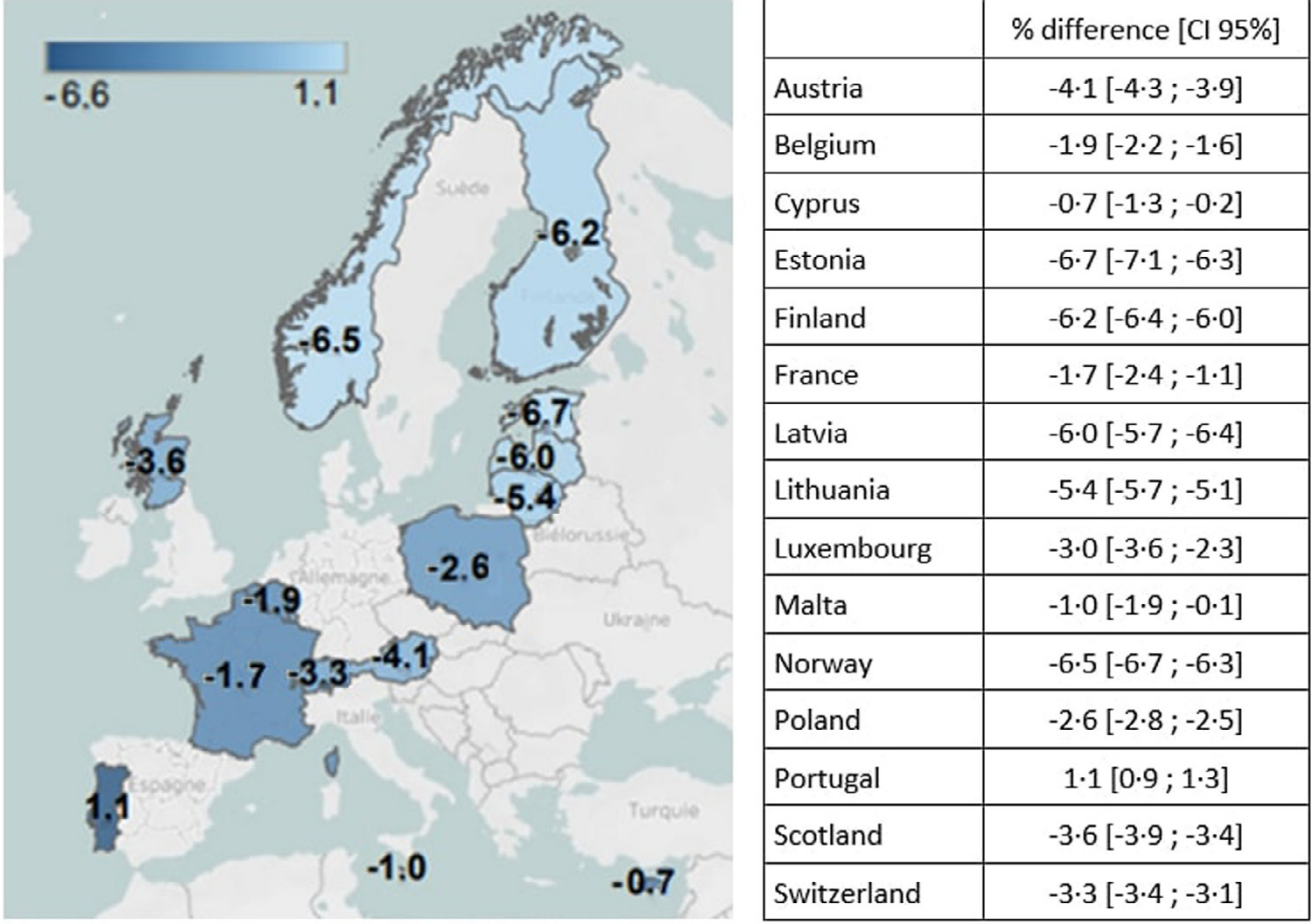
Difference in prevalence of SGA between international and national birthweight charts This map shows the geographic pattern of differences in SGA prevalence depending on the use of international compared to national birthweight charts, with the lightest blue color denoting countries where differences between the charts are most pronounced. Differences are largest in the north of Europe where international charts give lower SGA prevalence than the national charts.

**Fig. 1b:**
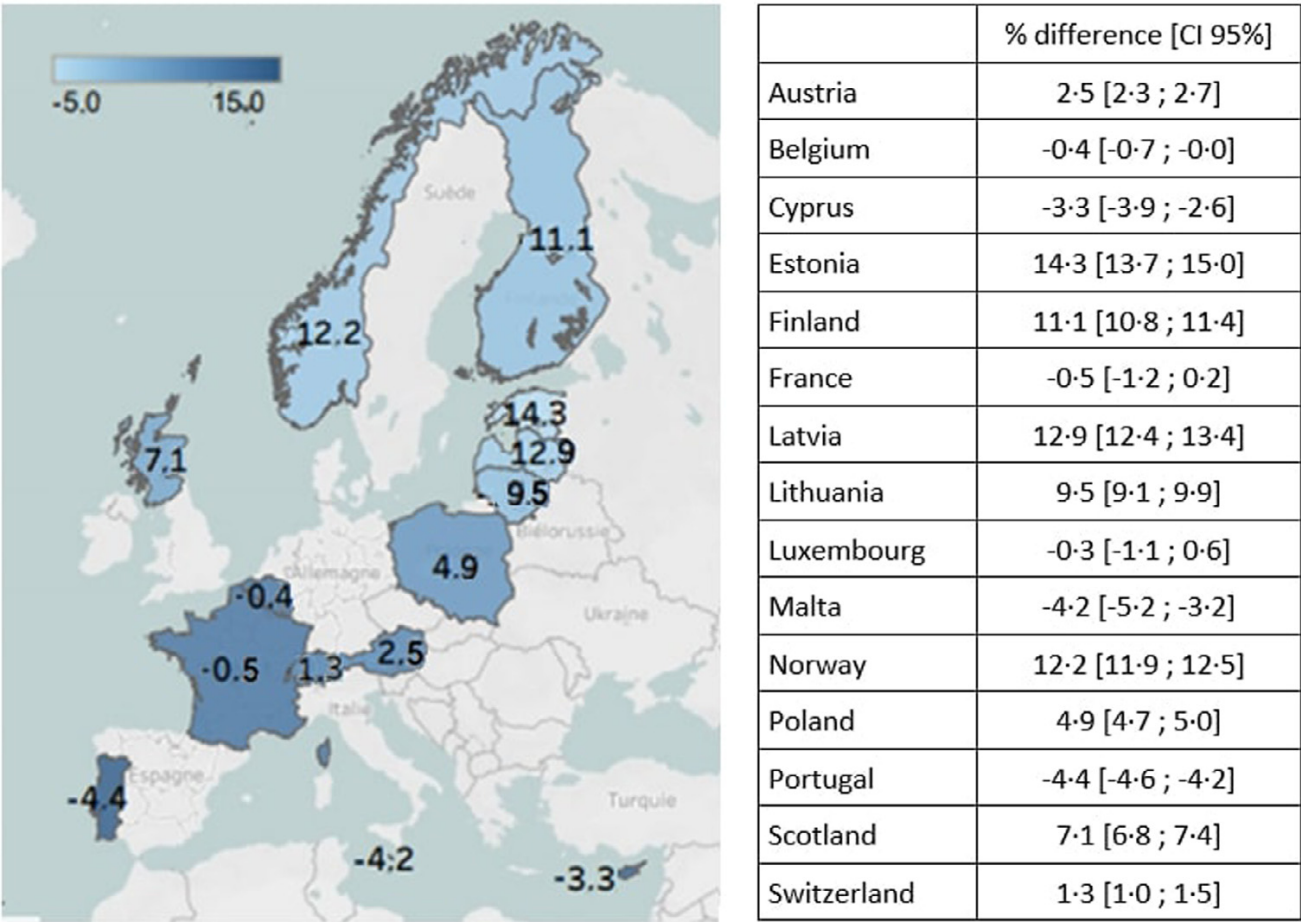
Difference in prevalence of LGA between international and national birthweight charts This map shows the geographic pattern of differences in LGA prevalence depending on use of international compared to national birthweight charts, with the lightest blue color denoting countries where the differences between the charts are most pronounced. Differences are largest in the north of Europe, where international charts give higher LGA prevalence than the national charts.

As shown in Table 3, 6.3% of infants in the overall sample were SGA according to both charts, 3.4% were SGA by national charts but AGA by the international charts, 73.2% were AGA by both charts, 5.4% were considered LGA by international charts but AGA by national charts and 10.7% were LGA according to both charts. Very few births were SGA according to international charts but AGA by national charts (0.2%) and AGA by international charts but LGA by national charts (0.9%); most of these births occurred in Portugal (99.4% and 56.2% respectively). Supplementary Table 2 provides these distributions by country. Infants considered AGA by both charts had mortality rates of 1.3, 0.6 and 1.9 per 1000 for stillbirth, neonatal death and perinatal death, respectively. These rates were highest for infants who were SGA according to both charts (8.5, 3.0 and 10.9 per 1000), followed by those SGA by national charts only (4.3, 1.4 and 5.6 per 1000). They were lowest for infants considered LGA by international charts only (0.7, 0.4 and 1.2 per 1000). These patterns were similar among term births.

**Table 3:** Risk of stillbirth, neonatal and perinatal death by birthweight (BW) classification for all births ≥ 33 weeks and term births.

Stillbirth	All births^1^	Stillbirths	Adjusted model^2^	All term births^1^	Term stillbirths	Adjusted model^2^
	N (%)	n (rate per 1000)	aOR [95% CI]	N (%)	n (rate per 1000))	aOR [95% CI]
SGA both	92 559 (6.3)	791 (8.5)	6.7 [6.1; 7.3]	85 035 (6.0)	434 (5.1)	5.9 [5.3; 6.6]
SGA international only	2 188 (0.2)	2 (0.9)	1.1 [0.3; 4.3]	2 163 (0.2)	2 (0.9)	1.8 [0.4; 7.3]
SGA national only	50 245 (3.4)	218 (4.3)	3.1 [2.7; 3.6]	45 744 (3.3)	101 (2.2)	2.2 [1.8; 2.8]
AGA both	1 079 324 (73.2)	1424 (1.3)	Reference	1 033 397 (73.4)	927 (0.9)	Reference
LGA national only	13 885 (0.9)	31 (2.2)	2.2 [1.5; 3.1]	10 745 (0.8)	5 (0.5)	0.8 [0.3; 1.8]
LGA international only	79 251 (5.4)	58 (0.7)	0.5 [0.4; 0.6]	79 216 (5.6)	58 (0.7)	0.7 [0.5; 0.9]
LGA both	158 005 (10.7)	196 (1.2)	0.9 [0.8; 1.1]	151 267 (10.8)	148 (1.0)	1.1 [0.9; 1.3]
Female	716 647 (48.6)	1 321 (1.8)	Reference	685 846 (48.7)	819 (1.2)	Reference
Male	758 810 (51.4)	1 399 (1.8)	1.0 [0.9; 1.1]	721 721 (51.3)	856 (1.1)	1.0 [0.9; 1.1]
Variance at country level			0.06 [0.03; 0.14]			0.08 [0.03; 0.20]


Neonatal death	Live births^1,3^	Neonatal deaths	Adjusted model^2^	Term live births^1^	Term neonatal deaths	Adjusted model^2^
	N (%)	n (rate per 1000)	aOR [95% CI]	N (%)	n (rate per 1000)	aOR [95% CI]
SGA both	64 082 (6.0)	194 (3.0)	5.4 [4.6; 6.4]	58 881 (5.8)	131 (2.2)	5.0 [4.1; 6.1]
SGA international only	2 186 (0.2)	1 (0.5)	1.3 [0.2; 9.6]	2 161 (0.2)	1 (0.5)	1.5 [0.2; 11.2]
SGA national only	39 271 (3.7)	56 (1.4)	2.2 [1.7; 2.9]	35 850 (3.6)	32 (0.9)	1.7 [1.2; 2.5]
AGA both	771 619 (72.8)	452 (0.6)	Reference	738 348 (73.0)	343 (0.5)	Reference
LGA national only	12 596 (1.2)	9 (0.7)	1.6 [0.8; 3.0]	10 211 (1.0)	1 (0.1)	0.3 [0.0; 1.9]
LGA international only	58 485 (5.5)	22 (0.4)	0.5 [0.4; 0.8]	58 450 (5.8)	22 (0.4)	0.7 [0.5; 1.1]
LGA both	112 046 (10.6)	77 (0.7)	1.1 [0.9; 1.5]	107 365 (10.6)	52 (0.5)	1.0 [0.8; 1.4]
Female	516 953 (48.8)	372 (0.7)	Reference	494 524 (48.9)	272 (0.6)	Reference
Male	543 332 (51.2)	439 (0.8)	1.1 [1.0; 1.3]	516 742 (51.1)	310 (0.6)	1.1 [0.9; 1.3]
Variance at country level			0.19 [0.07; 0.49]			0.16 [0.06; 0.42]

Perinatal death	All births^1,3^	Perinatal deaths	Adjusted model^2^	All term births^1^	Term perinatal deaths	Adjusted model^2^
	N (%)	n (rate per 1000))	aOR[95% CI]	N (%)	n (rate per 1000)	aOR [95% CI]
SGA both	64 590 (6.1)	702 (10.9)	6.1 [5.6; 6.7]	59 161 (5.8)	411 (6.9)	5.5 [4.9; 6.2]
SGA international only	2 188 (0.2)	3 (1.4)	1.1 [0.4; 3.6]	2 163 (0.2)	3 (1.4)	1.7 [0.6; 5.4]
SGA national only	39 436 (3.7)	221 (5.6)	2.7 [2.3; 3.1]	35 932 (3.6)	114 (3.2)	2.1 [1.7; 2.6]
AGA both	772 613 (72.7)	1446 (1.9)	Reference	738 987 (73.0)	982 (1.3)	Reference
LGA national only	12 618 (1.2)	31 (2.5)	1.7 [1.2; 2.4]	10 215 (1.0)	5 (0.5)	0.5 [0.2; 1.2]
LGA international only	12 618 (5.5)	72 (1.2)	0.6 [0.4; 0.7]	58 500 (5.8)	72 (1.2)	0.8 [0.6; 1.0]
LGA both	112 174 (10.6)	205 (1.8)	1.0 [0.8; 1.1]	107 461 (10.6)	148 (1.4)	1.0 [0.8; 1.2]
Female	517 853 (48.8)	1 272 (2.5)	Reference	495 080 (48.9)	828 (1.7)	Reference
Male	544 301 (51.2)	1 408 (2.6)	1.1 [1.0; 1.2]	517 339 (51.1)	907 (1.8)	1.1 [1.0; 1.2]
Variance at country level			0.09 [0.04; 0.22]			0.09 [0.04; 0.21]

NOTE: (1) ≥ 33 to ≤42 completed weeks of gestation (2) Model adjusted on fetal sex and with a supplementary level for the country (3) Births with data on perinatal death.

In mixed effects models adjusted for sex, infants classified as SGA by both charts faced highest mortality risks (aOR for perinatal death: 6.1 [5.6; 6.7]) compared to infants who were AGA according to both charts. Infants considered SGA by the national chart but AGA by the international chart also had increased risks of mortality (aOR for perinatal death: 2.7 [2.3; 3.1]). Being classified as LGA by the international chart only was associated with lower risks (aOR for perinatal death: 0.6 [0.4; 0.7]). Finally, being LGA according to both charts was not associated with an increased risk of mortality. Models for stillbirths and neonatal mortality yielded similar results, as did analyses restricted to term births.

## 4 Discussion

### 4.1 Main findings

Our results showed marked discordance between international and national charts for identifying SGA and LGA infants in European countries. Using national charts led to about 10% of infants being classified as SGA and LGA in all the countries, as expected. In contrast, applying international charts led to wide between-country variation from 3.0% to 10.1% for SGA and from 8.0% to 24.9% for LGA, following a geographic pattern of higher SGA prevalence in the south and higher LGA prevalence in the north. Compared to infants considered AGA by both charts, those reclassified from SGA to AGA using the international charts were at 2.7 (2.3 to 3.1) increased risk of perinatal death, whereas those reclassified from AGA to LGA using the international chart were at reduced risk 0.6 (0.4 to 0.7). Very few infants were reclassified from AGA to SGA using the international charts. Taken together, these results do not provide support for the use of international birthweight charts in Europe.

### 4.2 Interpretation

Intergrowth-21st international charts for intrauterine growth monitoring were published in 2014, but their application in daily practice is an on-going debate. Multiple single-country studies have compared Intergrowth-21st's newborn charts with national charts. Similar to our results, local birthweight charts[33], [34], [35] as well as Gardosi's customised model[36,37] have found that using Intergrowth-21st yielded a lower prevalence of SGA and a higher prevalence of LGA than national or customised charts. We add to this literature by showing that the differences in the prevalence of SGA and LGA when using international charts varied greatly between European countries and followed a geographic gradient from north to south. Our results support the position that population anthropometric characteristics should be considered in growth monitoring [8,[37], [38], [39]].

Our results also corroborate studies comparing mortality risks using international versus local or customised charts. Francis et al. found that being SGA by customised charts alone led to higher risks of stillbirth and adverse neonatal outcomes [36], and a Canadian study showed that detection rates for their composite mortality and morbidity outcome were higher among newborns considered SGA according to their local chart than among SGA according to Intergrowth-21st [33]. A Swedish study revealed that the risk of perinatal mortality was significantly increased up to the 35th percentile of the Intergrowth-21st chart but only up to the 15th percentile of their local chart [37]. We found that infants classified as SGA according to national charts, but considered AGA by the international chart, had an over two-fold increased risk of perinatal death when compared to those AGA by both charts. Since the national charts’ tenth percentile was higher than the international chart for all countries except Portugal, and mortality decreases linearly with weight percentile to an optimum which has been shown to be higher than the mean [8], an elevated risk in this group could be expected. However, the magnitude of the increased risk is of concern given the proportion and unequal geographic distribution of reclassified infants: 3.4% of the overall sample and over 6% in Estonia and Norway. Ideally, we would compare the performance of the charts in terms of sensitivity and specificity, however there is no consensual gold-standard as all current definitions of fetal growth restriction include at least one criterion based on a weight percentile defined in relation to a growth chart [2,[40], [41], [42]].

Infants reclassified as LGA according to the international chart had significantly lower risks of mortality than those AGA by both charts and represented about 10% or more of the births in the Nordic and Baltic countries. Infants considered LGA by both charts were not at higher risk for any of the outcomes compared to AGA infants according to both charts. This result differed from what was expected but may be explained by the fact that the association between excessive growth and mortality or morbidity has previously been investigated using absolute weights, over 4000 or 4500 gs, rather than percentiles[7,43]. Using a higher percentile cutoff, such as the 97th, may be more appropriate for capturing the mortality risks associated with LGA and should be explored in further studies. Our models also confirmed the well-documented increased risk of neonatal mortality among boys; risk of stillbirth did not differ which is in line with some recent studies showing no sex differences in overall stillbirth rates [44].

We derived national charts based on the customised model, which uses Hadlock's fetal growth model. This approach has been previously used to derive country-specific charts[16,21,22] and allowed for consistency across countries and provided proportions of SGA and LGA births in line with expectations. However, it differed from the methodology used in the Intergrowth project and from other birthweight charts. Differences in these charts occur primarily at preterm gestations because birthweight charts include preterm infants with abnormal growth and therefore preterm percentiles are generally lower [45,46]. Differences in the classification of preterm births do not explain our findings, however, as our results were similar when the sample was restricted to term births only. Results from other studies comparing Intergrowth with national curves have been similar for both types of national charts [33,47].

In our observational study of birthweight, we can only measure the differences between international and national newborn charts for identifying births facing higher risks of perinatal mortality. However, our study is in line with research on charts of ultrasound measures (in particular, abdominal circumference) or estimated fetal weight, showing a lower proportion of fetuses with growth parameters under the tenth percentile as well as lower sensitivity of the Intergrowth 21st charts for identifying growth restricted fetuses during pregnancy compared to local or customised charts [47], [48], [49]. The population used to build the Intergrowth 21st charts are the same for the fetal and the newborn charts, and therefore concerns about this reference population apply more broadly. Antenatal screening using charts that are not adapted to the population could lead to failure to identify SGA fetuses and insufficient monitoring of high risk pregnancies, while over-identification of LGA fetuses could increase iatrogenic interventions, parental stress and healthcare costs [50]. Accurate identification of fetuses and newborns at risk is vital to enable appropriate antenatal monitoring and interventions that prevent stillbirth and neonatal morbidity[51], [52], [53] and to guide management after birth.

### 4.3 Strengths and limitations

This study's strengths are its use of population data from a diverse sample of countries, enabling assessment of the consequences of using international charts on comparisons of sub-optimal growth in Europe. By cumulating data from many countries and over several years, we were able to attain a sample sufficient for investigating fetal and neonatal mortality which are rare events. Limitations are the absence of data on other environmental and maternal characteristics which influence growth. More research is warranted on the factors that influence birthweight in Europe, including the cultural and environmental context (diet or pollutants, for example), physiological characteristics (maternal and paternal height, genetic factors) and risk factors for sub-optimal growth (maternal smoking, maternal obesity and underweight, older maternal age, social disadvantage) to assess their relevance for antenatal and neonatal growth monitoring. Data were from 2010 to 2014, but birthweight as an indicator is stable over time [26,54], the current rate of change in perinatal mortality in Europe is low[26] and the question of whether universal charts should be applied is not time-bound. Because data come from diverse routine sources, we were not able to clearly assess methods for determining gestational age, although countries in Europe all provide early prenatal care, with widespread use of dating ultrasounds [55]. Finally, although we had large samples from a geographically diverse sample, we were not able to study mortality risks stratified at the country-level because the number of deaths was too small in some countries.

## 5 Conclusion

Our results do not provide support for the use of the Intergrowth 21st international charts for defining SGA and LGA at birth in Europe as this could lead to the underestimation of infants with SGA and overestimation of LGA in some countries. Their use for comparative surveillance and research is also problematic as differences in SGA and LGA prevalence between countries were influenced strongly by population anthropometric characteristics and cannot be interpreted as reflecting variations in perinatal health risks.

## Funding statement

The Euro-Peristat network receives funding from the European Commission as part of the InfAct (Information for Action) Joint Action (Consumers, Health, Agriculture and Food Executive Agency (CHAFEA) Grant n° 801,553). This work was conducted as part of the ConcePTION project which has received funding from the Innovative Medicines Initiative 2 Joint Undertaking under grant agreement No 821,520. This Joint Undertaking receives support from the European Union's Horizon 2020 research and innovation program and EFPIA. Alice HOCQUETTE was supported by a PhD grant from EHESP. While the research leading to these Results was conducted as part of the ConcePTION consortium, this paper only reflects the personal views of the stated authors.

## Authors’ contribution

AH and JZ had full access to all of the data in the study and take responsibility for the integrity of the data and the accuracy of the data analyses.

Study concept and design: JZ, AH

Data acquisition and interpretation AH, MD, KS, RW, KK, SB, TR, TK, LS, AL, IZ, SA, JK, HB, MG, JI, BB, MG, JZ

Drafting of the manuscript: AH, JZ, KS, RW, KK, SB, TR

Critical revision of the manuscript for important intellectual content and approval of final version of the manuscript: AH, MD, KS, RW, KK, SB, TR, TK, LS, AL, IZ, SA, JK, HB, MG, JI, BB, MG, JZ

Data management and statistical analysis AH, JZ, MD

## Data sharing statement

Individual participant data will not be available to others. Data on birthweight percentiles modeled for each participating country are available upon request from the authors.

## Declaration of interest

No conflict of interest to disclose.

## Appendix Supplementary materials

Image, image 1

Image, application 2

## Footnotes

Supplementary material associated with this article can be found in the online version at doi: https://doi.org/10.1016/j.lanepe.2021.100167.
