# Supplementary figures and images for: International versus national growth charts for identifying small and large-for-gestational age newborns: A population-based study in 15 European countries
Source: Lancet Reg Health Eur. 2021 Jul 15;8:100167. doi: 10.1016/j.lanepe.2021.100167 (PMC8454535; doi:10.1016/j.lanepe.2021.100167)

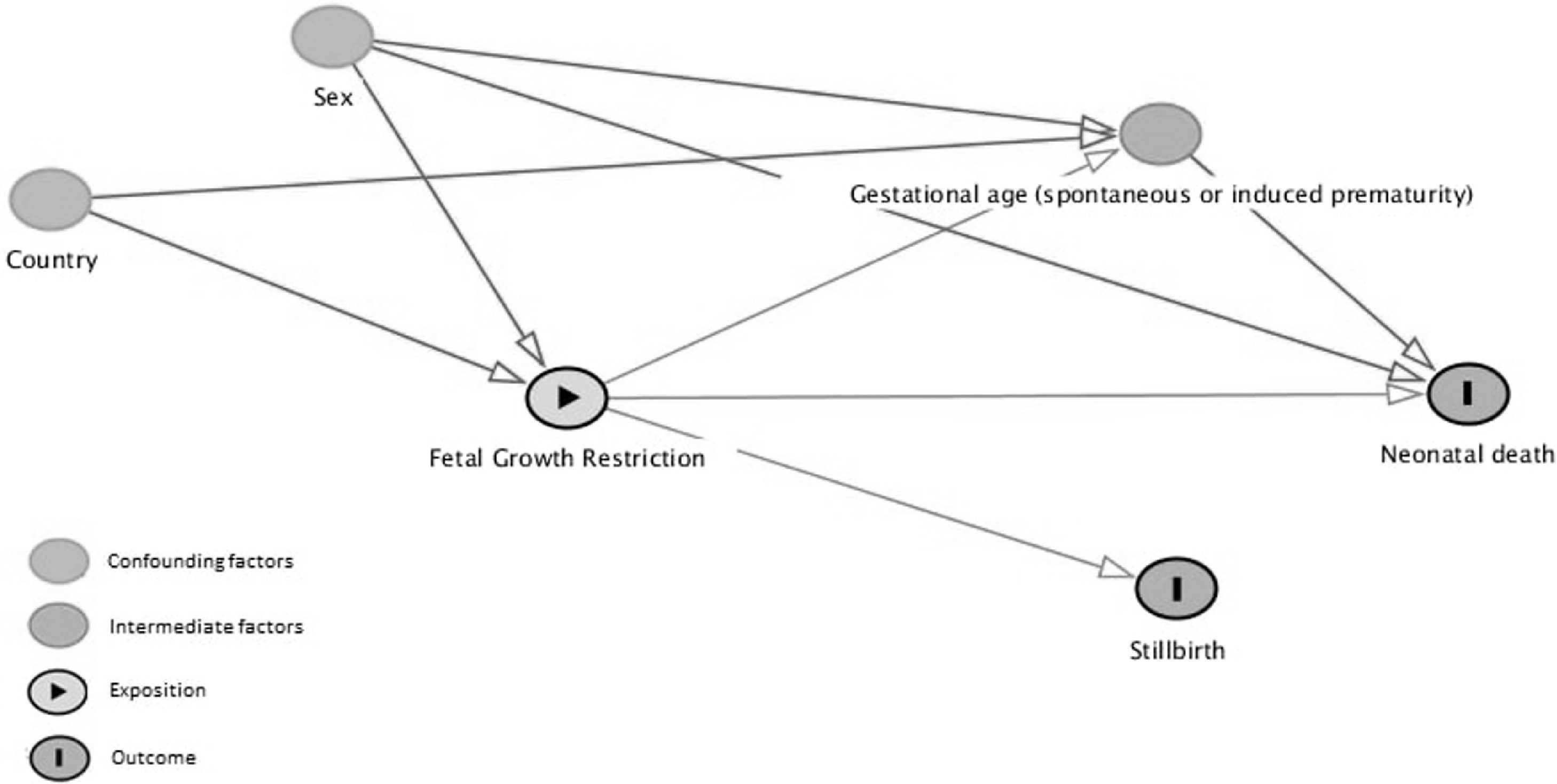

Supplement: Supplementary file 1 [file mmc1.jpg]
