# Supplementary material for: International versus national growth charts for identifying small and large-for-gestational age newborns: A population-based study in 15 European countries
Source: Lancet Reg Health Eur. 2021 Jul 15;8:100167. doi: 10.1016/j.lanepe.2021.100167 (PMC8454535; doi:10.1016/j.lanepe.2021.100167)
Supplement: Supplementary file 2 [file mmc2.docx]

Supplementary materials

**Supplementary Table 1: Countries’ mean birthweight and coefficient of variation at 40 weeks of gestation used to create national charts**

| **Country** | **Boys** | | **Girls** | |
| --- | --- | --- | --- | --- |
|  | **Mean birthweight at 40 weeks of gestation**  **(m_C_)** | **Coefficient of variation at 40 weeks of gestation**  **(s_C_)** | **Mean birthweight at 40 weeks of gestation**  **(m_C_)** | **Coefficient of variation at 40 weeks of gestation**  **(s_C_)** |
| AUSTRIA | 3594 | 11·5 | 3443 | 11·7 |
| BELGIUM | 3532 | 11·7 | 3388 | 12·0 |
| CYPRUS | 3495 | 11·8 | 3346 | 11·6 |
| ESTONIA | 3752 | 11·4 | 3596 | 11·7 |
| FINLAND | 3698 | 11·5 | 3559 | 11·6 |
| FRANCE ENP | 3511 | 11·7 | 3378 | 11·7 |
| LATVIA | 3734 | 12·0 | 3570 | 11·9 |
| LUXEMBOURG | 3552 | 11·1 | 3409 | 11·7 |
| MALTA | 3492 | 11·5 | 3341 | 11·2 |
| NORWAY | 3704 | 11·6 | 3583 | 11·7 |
| POLAND | 3604 | 12·3 | 3441 | 12·3 |
| PORTUGAL | 3439 | 11·9 | 3310 | 11·7 |
| SCOTLAND | 3622 | 12·2 | 3478 | 12·3 |
| SWITZERLAND | 3571 | 11·5 | 3416 | 11·5 |
| LITHUANIA | 3690 | 11·8 | 3537 | 11·8 |

**Supplementary Table 2: SGA. AGA and LGA distribution according to both national and international charts in each country**

|  |  | **SGA both** | **SGA international only** | **SGA national only** | **AGA both** | **LGA national only** | **LGA international only** | **LGA both** | **Total** |
| --- | --- | --- | --- | --- | --- | --- | --- | --- | --- |
| AUSTRIA | n | 9031 | 0 | 6282 | 115579 | 865 | 4713 | 16940 | 153410 |
|  | % | 5·9 | 0·0 | 4·1 | 75·3 | 0·6 | 3·1 | 11·0 | 100·0 |
| BELGIUM | n | 5905 | 0 | 1339 | 55292 | 1000 | 741 | 7711 | 71988 |
|  | % | 8·2 | 0·0 | 1·9 | 76·8 | 1·4 | 1·0 | 10·7 | 100·0 |
| CYPRUS | n | 1556 | 13 | 164 | 15625 | 737 | 74 | 2121 | 20290 |
|  | % | 7·7 | 0·1 | 0·8 | 77·0 | 3·6 | 0·4 | 10·5 | 100·0 |
| ESTONIA | n | 855 | 0 | 1901 | 18476 | 3 | 4061 | 2988 | 28284 |
|  | % | 3·0 | 0·0 | 6·6 | 65·3 | 0·0 | 14·4 | 10·6 | 100·0 |
| FINLAND | n | 4514 | 0 | 7090 | 76563 | 40 | 12795 | 13608 | 114610 |
|  | % | 3·9 | 0·0 | 6·2 | 66·8 | 0·0 | 11·2 | 11·9 | 100·0 |
| FRANCE | n | 1284 | 0 | 253 | 11223 | 227 | 157 | 1395 | 14539 |
|  | % | 8·8 | 0·0 | 1·6 | 77·2 | 1·6 | 1·1 | 9·6 | 100·0 |
| LATVIA | n | 1583 | 0 | 2357 | 26138 | 1 | 5040 | 4047 | 39166 |
|  | % | 4·0 | 0·0 | 6·0 | 66·7 | 0·0 | 12·9 | 10·3 | 100·0 |
| LITHUANIA | n | 2490 | 0 | 3069 | 39762 | 23 | 5440 | 6240 | 57024 |
|  | % | 4·4 | 0·0 | 5·4 | 69·7 | 0·0 | 9·5 | 10·9 | 100·0 |
| LUXEMBOURG | n | 830 | 0 | 382 | 9852 | 180 | 147 | 1463 | 12854 |
|  | % | 6·5 | 0·0 | 3·0 | 76·7 | 1·4 | 1·1 | 11·4 | 100·0 |
| MALTA | n | 687 | 0 | 82 | 6032 | 337 | 1 | 845 | 7984 |
|  | % | 8·6 | 0·0 | 1·0 | 75·6 | 4·2 | 0·0 | 10·6 | 100·0 |
| NORWAY | n | 4562 | 0 | 7537 | 77309 | 19 | 14239 | 12937 | 116603 |
|  | % | 3·9 | 0·0 | 6·5 | 66·3 | 0·0 | 12·2 | 11·1 | 100·0 |
| POLAND | n | 26685 | 0 | 10556 | 295488 | 1040 | 20559 | 44436 | 398764 |
|  | % | 6·7 | 0·0 | 2·7 | 74·1 | 0·3 | 5·2 | 11·1 | 100·0 |
| PORTUGAL | n | 15707 | 2175 | 284 | 136807 | 7803 | 44 | 14193 | 177013 |
|  | % | 8·9 | 1·2 | 0·2 | 77·3 | 4·4 | 0·0 | 8·0 | 100·0 |
| SCOTLAND | n | 7018 | 0 | 3903 | 76944 | 92 | 7746 | 12088 | 107791 |
|  | % | 6·5 | 0·0 | 3·6 | 71·4 | 0·1 | 7·2 | 11·2 | 100·0 |
| SWITZERLAND | n | 9852 | 0 | 5046 | 118234 | 1518 | 3494 | 16993 | 155137 |
|  | % | 6·4 | 0·0 | 3·3 | 76·2 | 1·0 | 2·3 | 11·0 | 100·0 |
| Total | n | 93512 | 2188 | 51784 | 1096494 | 13890 | 82070 | 160738 | 1500676 |
|  | % | 6·2 | 0·2 | 3·5 | 73·1 | 0·9 | 5·5 | 10·7 | 100·0 |

**Appendix A: list of data sources**

| **Country** | **Data source** |
| --- | --- |
| Austria | Vital Statistics |
| Belgium | Fédération Wallonie-Bruxelles / Région wallonne |
| Cyprus | Medical Birth Register of Public Maternity Units |
| Estonia | Estonian Medical Birth Registry; Estonian Registry on Causes of Death |
| Finland | THL Medical Birth Register |
| France | National Perinatal Survey |
| Latvia | Medical Birth Register |
| Lithuania | Medical Date of Births |
| Luxembourg | Système de Surveillance de la Santé |
| Malta | National Obstetrics Information System. Directorate for Health Information and Research |
| Norway | Medical Birth Registry of Norway |
| Poland | Central Statistical Office |
| Portugal | National Statistics (INE - Instituto Nacional de Estatística) |
| Scotland | National Records for Scotland and Public Health Scotland: vital event and maternal hospital discharge data |
| Switzerland | BEVNAT. statistics of natural population change |
